# Adherence to Mediterranean Diet of Breastfeeding Mothers and Fatty Acids Composition of Their Human Milk: Results From the Italian MEDIDIET Study

**DOI:** 10.3389/fnut.2022.891376

**Published:** 2022-06-02

**Authors:** Matteo Di Maso, Francesca Bravi, Monica Ferraroni, Carlo Agostoni, Simone R. B. M. Eussen, Tamás Decsi, Pasqua A. Quitadamo, Paola Tonetto, Chiara Peila, Claudio Profeti, Guglielmo Salvatori, Iwona Kazmierska, Adriano Decarli, Elisabetta Vacca, Enrico Bertino, Bernd Stahl, Guido E. Moro

**Affiliations:** ^1^Department of Clinical Sciences and Community Health, Branch of Medical Statistics, Biometry and Epidemiology “G.A. Maccacaro”, Università degli Studi di Milano, Milan, Italy; ^2^Fondazione IRCCS Ca’ Granda, Ospedale Maggiore Policlinico, Milan, Italy; ^3^Pediatric Intermediate Care Unit, Fondazione IRCCS Ospedale Cà Granda-Ospedale Maggiore Policlinico, Milan, Italy; ^4^Department of Clinical Science and Community Health, Università degli Studi di Milano, Milan, Italy; ^5^Danone Nutricia Research, Utrecht, Netherlands; ^6^Department of Paediatrics, University of Pécs, Pécs, Hungary; ^7^Neonatology and Neonatal Intensive Care Unit, IRCCS Casa Sollievo della Sofferenza, San Giovanni Rotondo, Italy; ^8^Neonatal Intensive Care Unit, City of Health and Science, University of Turin, Turin, Italy; ^9^Azienda Ospedaliera Universitaria Meyer di Firenze, Florence, Italy; ^10^Neonatal Intensive Care Unit, IRCCS Ospedale Pediatrico Bambino Gesù, Rome, Italy; ^11^Neonatal Intensive Care Unit, Ospedale Buccheri La Ferla Fatebenefratelli, Palermo, Italy; ^12^Department of Public Health and Pediatrics, Neonatal Intensive Care Unit, Università degli Studi di Torino, Turin, Italy; ^13^Department of Chemical Biology and Drug Discovery, Utrecht Institute for Pharmaceutical Sciences, Faculty of Science, Utrecht University, Utrecht, Netherlands; ^14^Italian Association of Human Milk Banks (AIBLUD), Milan, Italy

**Keywords:** maternal nutrition, Mediterranean diet, breastfeeding, lipids, fatty acids profile

## Abstract

**Background and Aims:**

The content of fatty acids (FA) in human milk may be influenced by maternal nutrition. We evaluated the role of a Mediterranean diet in influencing the content of FA in human milk among 282 breastfeeding mothers participating in the MEDIDIET study.

**Materials and Methods:**

Mediterranean Diet Score (MDS), a composite index, was used to evaluate adherence to the Mediterranean diet. It includes 9 components (i.e., vegetables, fruit, cereals, legumes, monounsaturated to saturated FA ratio – MUFA/SFA, fish, dairy products, meat, and alcohol) and therefore potentially ranges from 0 (no adherence) to 9 (complete adherence) points. None of the mothers obtained the highest score due to the low alcohol consumption in the study population. Mothers were categorized in approximate tertiles of adherence: 0–3 (34.4%), 4 (26.2%), and 5–8 points (39.4%). The mean content of FA across categories of MDS was compared using ANOVA and test for trend.

**Results:**

A high adherence of breastfeeding mothers to the Mediterranean diet was associated with lower content of SFA in human milk (42.58 ± 4.36 for MDS = 0–3, 42.58 ± 4.89 for MDS = 4, and 40.92 ± 5.22% of fats for MDS = 5–8 points; *p* ANOVA and *p* for trend = 0.02). Conversely, a high adherence was associated with higher content of MUFA in human milk (43.27 ± 4.27 for MDS = 0–3, 43.27 ± 4.47 for MDS = 4, and 45.24 ± 5.22% of fats for MDS = 5–8 points; *p* ANOVA and *p* for trend < 0.01), ω-3 FA (1.07 ± 0.25 for MDS = 0–3, 1.22 ± 0.49 for MDS = 4, and 1.31 ± 0.51% of fats for MDS = 5–8 points; *p* ANOVA and *p* for trend < 0.01), and the major types of ω-3 FA (i.e., α-linolenic acid – ALA, eicosapentaenoic acid – EPA, docosahexaenoic acid – DHA, docosapentaenoic acid – DPA). These associations were mainly driven by the adherence to the vegetables, MUFA/SFA, fish, and dairy products components of the Mediterranean diet.

**Conclusion:**

A high adherence to the Mediterranean diet was associated with human milk showing a lower content of SFA and higher content of MUFA and ω-3 FA, including DHA. The Mediterranean diet may contribute in human milk production with higher content of specific FA which is directly involved in infant’s neural and visual development, as reported by previous studies.

## Introduction

Human milk is a mixture of nutritive and bioactive compounds (e.g., nutrients, hormones, antibodies, immune and stem cells, digestive enzymes, and macrophages) that contribute to the growth and development of the infant (1). Maternal dietary habits may influence the composition of human milk, especially the fatty acids (FA) content (2). In particular, it has been shown that maternal dietary habits modify the content of polyunsaturated FA (PUFA) in human milk and, to a lesser extent, the content of saturated FA (SFA) and monounsaturated FA (MUFA) (3).

Many studies investigated the relationship between maternal nutrition and the composition of human milk according to the intake of single foods or nutrients (4), while a few studies evaluated the role of *a posteriori* dietary patterns (5–8). In particular, only one study from China (7) and one of our previous analyses (5) investigated the adherence of breastfeeding mothers to *a posteriori* dietary patterns concerning the content of FA in human milk. Two additional studies evaluated the relationship between the adherence of breastfeeding mothers to *a posteriori* dietary patterns and the composition of their human milk focusing on macronutrients, without taking into account FA (6, 8).

The traditional Mediterranean diet is a plant-oriented dietary pattern characterized by a high intake of minimally processed foods, fresh fruits, bread and other cereals (generally minimally refined), potatoes, legumes, and nuts; a moderate intake of dairy products (mostly cheese and yogurt); a moderate to low intake of fish and poultry; a low intake of red meat; a high intake of olive oil (especially virgin and extra-virgin olive oils) used as the main source of fat; and a moderate intake of wine, consumed with meals (9). Adherence to the Mediterranean diet has been associated with a range of health benefits, including a reduced risk of cardiovascular diseases, diabetes, metabolic syndrome, overweight and obesity, several cancer types, as well as a lower disease-specific and overall mortality (10–13). However, no study has investigated so far the association between maternal adherence to the Mediterranean diet, measured by one of the proposed scores, and the content of FA in human milk.

The Italian MEDIDIET study aims to evaluate the relationship between the habitual diet of breastfeeding mothers and the nutritional composition, as well as oxidative/anti-oxidative properties of their milk (14). The role of the Mediterranean diet in influencing human milk composition is at the core of the MEDIDIET study. Thus, we present here the FA human milk profile of breastfeeding mothers participating in the MEDIDIET study according to their adherence to the traditional Mediterranean diet.

## Materials and Methods

### Study Design and Participants

Between 2012 and 2014, the MEDIDIET study was carried out in five Italian maternity wards (i.e., Turin, Florence, Rome, San Giovanni Rotondo – SGR, and Palermo) enrolling 300 healthy breastfeeding mothers. Information on study design, inclusion criteria, maternal diet assessment, human milk collection, and analysis of human milk samples has been provided in detail elsewhere (14). Briefly, mothers were 25–41 years old and gave birth to healthy term infants. In particular, inclusion criteria for mothers were

• not chronically diseased (i.e., free of diabetes, autoimmune diseases, cardiovascular disease, renal disease, and hypertension);
• seronegative for hepatitis B, hepatitis C, and human immunodeficiency viruses;
• non-smokers during pregnancy and lactation;
• non-abusers of drugs or alcohol;
• non-severely obese (i.e., BMI < 35 kg/m2);
• not on a restricted diet.

Inclusion criteria for infants were:

• born at ≥37 gestational weeks;
• weight range of 2,500–4,500 g;
• body length range of 46–56 cm;
• exclusively breastfed from birth to the day of human milk collection (i.e., 6 ± 1 week postpartum).

All mothers signed an informed consent form to participate in the study. The Ethics Committee of participating hospitals approved the study (protocol number: 31060 MD) as per the ethical standards of the Declaration of Helsinki.

### Human Milk Collection and Analysis

On the day of human milk collection (i.e., 6 ± 1 weeks postpartum), mothers provided a sample of their foremilk (30–50 ml) expressed in the morning after breakfast and before lunch, using a breast pump. The time elapsed since the end of the previous breastfeeding session ranged from 1 to 3 h. The freshly expressed human milk was stirred and divided in sterile 10 ml tubes, overlaid with nitrogen gas to avoid oxidation, and then stored at −70°. Human milk analyses included macronutrient composition (i.e., protein, lactose, fat, and energy density), oxidative parameters, and complete FA profile as reported in Moro et al. (14). Briefly, FAs with a chain length between 4 and 24 carbon atoms of total lipids of human milk samples were analyzed as methyl esters by capillary gas chromatography with flame ionization detection (“GC-FID”) according to the method proposed by Beermann et al. (15). In addition, the lipid content was analyzed using the method outlined by Lucas et al. (16) and Jones et al. (17). In particular, the FA analyzed included SFA, MUFA, trans-FA (TFA), branched-chain FA (BFA), and PUFA. Peak identification of SFA, MUFA, TFA, and both ω-3 and ω-6 FA methyl esters were verified and calibrated by comparison with authentic standards (NuChek Prep; Elysian, MN, United States: GLC-463, GLC-473, GLC-642, GLC-643, GLC-674, and Sigma-Aldrich Ltd., St. Louis, MO, United States: Supelco PUFA3). The standard mix of BFA methyl esters was originally analyzed by Danone Nutricia Research, Utrecht, the Netherlands. Lastly, FA concentrations were recorded as% of fats.

### Assessment of Maternal Diet and Adherence to the Mediterranean Diet

A validated and reproducible food frequency questionnaire (FFQ) (18–20) was administered by trained interviewers to investigate the habitual diet of mothers (from partum to the day of human milk collection). The FFQ included 7 sections to collect information on the weekly intake of 78 food items, recipes, and beverages as follows: (1) milk, hot beverages, and sweeteners; (2) typical Italian first courses (e.g., pasta, risotto, gnocchi, lasagne); bread, and cereals; (3) second courses (including meat, fish, and cheese); (4) side dishes (e.g., vegetables); (5) fruits; (6) sweets, desserts, and soft drinks; and (7) alcoholic beverages. The serving size was defined in “natural” units (e.g., 1 cup of milk, 1 coffee spoon of sugar, 1 egg, 1 apple) or as smaller, on average, or larger than a standard Italian serving (e.g., 80 g of pasta, 100 g of mixed salad, 175 g of potatoes, and 150 g of beef). Occasional intakes (i.e., less than once a week, but greater than once per month) were coded as 0.5 servings per week. Dietary data collected using the FFQ were used to estimate maternal intake of energy and nutrients using an Italian food composition database (21). In this computation, we weighted the fat composition of each food or recipe according to information on the type of fat used for cooking or as dressing.

To evaluate the adherence of the maternal diet to the Mediterranean one, we used an *a priori* score (i.e., Mediterranean Diet Score – MDS) developed by Trichopoulou et al. (22, 23). According to the proposed methodology, the MDS includes 9 dietary components: (1) vegetables; (2) fruit; (3) cereals (including bread and potatoes); (4) legumes; (5) MUFA to SFA ratio (MUFA/SFA) as a proxy of olive oil consumption (23); (6) fish; (7) dairy products (including milk); (8) meat (including poultry, red and processed meat); (9) and alcohol. Each of the 9 components is assigned a score of 0 or 1 using fixed or median intakes as cutoffs according to the component considered. In particular, for components more frequently consumed in the Mediterranean diet (i.e., vegetables, fruit, cereals, legumes, fish, and MUFA/SFA), a score of 1 is given if the individual intake is greater or equal to the median, and 0 if the individual intake is lower than the median; for components less frequently consumed (i.e., dairy products and meat), a score of 1 is given if the individual intake is lower than the median, and 0 if the individual intake is greater or equal to the median. For alcohol component, a score of 1 is given for an individual consumption ranging from 5 to less than 25 g of ethanol/day, and 0 if the individual consumption is outside this range. The MDS is obtained by adding up the 9 component scores, and therefore, it potentially ranges from 0 (no adherence) to 9 (complete adherence) points.

### Statistical Analyses

The FA content was not available for 18 human milk samples due to insufficient milk volume, thus leaving 282 samples for the present analysis. We categorized the MDS in approximate tertiles: 0–3 points (*n* = 97; 34.4%) for a low adherence, 4 points (*n* = 74; 26.4%) for a medium adherence, and 5–8 points (*n* = 111; 39.4%) for a high adherence to the Mediterranean diet. None of the mothers obtained the theoretical highest score (i.e., MDS = 9) mainly due to the common Italian behavior of excluding or substantially reducing alcohol from the diet during the lactation period (24). We compared the mean content of FA in human milk according to categories of MDS using ANOVA adjusting for the ratio between the maternal energy intake and maternal pre-pregnancy weight (kcal/day/kg). In addition, we used the test for trend to evaluate the linear relationship of the mean contents of FA in human milk across categories of MDS. All analyses were conducted using R version 4.0.5.

## Results

### Description of the Study Population and Dietary Intake of Breastfeeding Mothers According to the Mediterranean Diet Score Components

Table 1 reports the demographic characteristics, pre-pregnancy BMI, and energy intake of the 282 breastfeeding mothers. Mothers had a mean age of 33 ± 4 years, a mean pre-pregnancy BMI of 22.3 ± 3.2 kg/m^2^, and a mean energy intake of 1947 ± 443 kcal/day.

**TABLE 1:** Distribution of 282 Italian breastfeeding mothers according to center, age, pre-pregnancy weight and BMI, and energy intake.

Variable	Descriptive statistic^a^
**Center**	
Turin	96 (34.0)
Florence	21 (7.5)
Rome	46 (16.3)
SGR	99 (35.1)
Palermo	20 (7.1)
Age (years)	28–38; 33 ± 4
Pre-pregnancy weight (kg)	50–71; 60 ± 9
Pre-pregnancy BMI (kg/m^2^)	18.8–26.2; 22.3 ± 3.2
Energy intake (kcal/day)	1485–2456; 1947 ± 443

*^a^Categorical variables are expressed as absolute frequency and relative frequency in parenthesis; continuous variables are expressed as 10th and 90th percentile range, and as mean ± standard deviation. SGR, San Giovanni Rotondo; BMI, body mass index. MEDIDIET study, 2012–2014.*

The distributions of MDS components (10th, 25th, 50th, 75th, and 90th percentile and mean ± standard deviation) are reported in Table 2. Overall, the median intakes (servings/week) of components more frequently consumed in the Mediterranean diet were 11.0 for vegetables, 15.0 for fruit, 22.0 for cereals, 1.0 for legumes, and 2.0 for fish; the median intake of the MUFA/SFA component was 1.24. The median intakes (servings/week) of components less frequently consumed in the Mediterranean diet were 11.0 for dairy products, and 8.0 for meat; the median intake for the alcohol component was 0.0 g ethanol/day.

**TABLE 2:** Distribution of components of the Mediterranean Diet Score.

MDS component	Percentile					Mean ± SD
	10th	25th	50th	75th	90th	
Vegetables (servings/week)	4.5	7.0	11.0	16.0	21.5	12.0 ± 7.0
Fruit (servings/week)	7.0	11.0	15.0	20.0	27.0	16.0 ± 8.5
Cereals (servings/week)	13.0	17.0	22.0	28.0	35.5	23.5 ± 10.0
Legumes (servings/week)	0.0	0.0	1.0	2.0	3.0	1.5 ± 1.5
MUFA/SFA	1.00	1.12	1.24	1.40	1.57	1.27 ± 0.23
Fish (servings/week)	0.5	1.0	2.0	2.0	3.0	1.5 ± 1.0
Dairy products (servings/week)	3.5	8.0	11.0	14.0	18.5	11.0 ± 5.5
Meat (servings/week)	5.0	6.5	8.0	10.0	12.5	8.5 ± 3.0
Alcohol (grams/day)	0.0	0.0	0.0	1.1	3.6	1.0 ± 2.5

*MDS, Mediterranean Diet Score; MUFA, monounsaturated fatty acids; SD, standard deviation; SFA, saturated fatty acids. MEDIDIET study, 2012–2014.*

### Content of Fatty Acids in Human Milk According to the Adherence of Breastfeeding Mothers to the Mediterranean Diet

High adherence to the Mediterranean diet was associated with a significantly lower content of SFA in human milk (*p* ANOVA = 0.02 and *p* for trend = 0.02; Table 3). In particular, the mean contents of SFA were 42.58 ± 4.36% of fats for mothers with low adherence to the Mediterranean diet (MDS = 0–3 points), 42.58 ± 4.89% of fats for mothers with medium adherence (MDS = 4 points), and 40.92 ± 5.22% of fats for mothers with high adherence (MDS = 5–8 points). Considering the major types of SFA, a high adherence of breastfeeding mothers to the Mediterranean diet was associated with a significantly lower content of palmitic acid (PA) and stearic acid (SA).

**TABLE 3:** Mean and standard deviation contents of selected fatty acids (expressed as% of fats) in human milk according to the adherence of breastfeeding mothers to the Mediterranean Diet Score (expressed approximately in tertiles).

FA in human milk	Total		MDS			*p*-value (ANOVA)^a^	*p*-value (trend)^a^
			0–3 points *n* = 97 (34.4)	4 points *n* = 74 (26.2)	5–8 points *n* = 111 (39.4)		
				
	Min-max	Mean ± SD	Mean ± SD	Mean ± SD	Mean ± SD		
SFA (% of fats)	27.89–56.11	41.92 ± 4.90	42.58 ± 4.36	42.58 ± 4.89	40.92 ± 5.22	*p* = 0.02	*p* = 0.02
CPA (% of fats)	0.08–0.51	0.24 ± 0.07	0.23 ± 0.08	0.25 ± 0.07	0.25 ± 0.07	*p* = 0.14	*p* = 0.13
CA (% of fats)	0.01–3.04	1.50 ± 0.42	1.43 ± 0.44	1.55 ± 0.42	1.53 ± 0.40	*p* = 0.11	*p* = 0.08
LAU (% of fats)	1.90–12.11	5.18 ± 1.92	4.99 ± 1.81	5.54 ± 2.14	5.11 ± 1.85	*p* = 0.15	*p* = 0.62
MA (% of fats)	2.11–11.89	5.45 ± 1.65	5.40 ± 1.58	5.69 ± 1.79	5.33 ± 1.62	*p* = 0.33	*p* = 0.80
PA (% of fats)	15.65–28.61	22.45 ± 2.40	23.18 ± 1.98	22.56 ± 2.43	21.75 ± 2.53	*p* < 0.01	*p* < 0.01
SA (% of fats)	3.26–9.27	5.81 ± 1.04	6.05 ± 1.00	5.71 ± 1.00	5.66 ± 1.06	*p* = 0.01	*p* < 0.01
MUFA (% of fats)	31.12–60.82	44.05 ± 4.79	43.27 ± 4.27	43.27 ± 4.47	45.24 ± 5.22	*p* < 0.01	*p* < 0.01
OA (% of fats)	26.23–56.63	39.29 ± 4.56	38.49 ± 4.00	38.61 ± 4.19	40.45 ± 5.03	*p* < 0.01	*p* < 0.01
EA (% of fats)	0.04–0.20	0.08 ± 0.02	0.07 ± 0.02	0.07 ± 0.02	0.08 ± 0.08	*p* = 0.01	*p* < 0.01
PUFA (% of fats)	9.03–24.32	13.59 ± 2.52	13.71 ± 2.52	13.71 ± 2.42	13.41 ± 2.61	*p* = 0.62	*p* = 0.35
ω-6 (% of fats)	8.14–23.10	12.39 ± 2.51	12.64 ± 2.51	12.49 ± 2.38	12.10 ± 2.58	*p* = 0.27	*p* = 0.11
LA (% of fats)	6.41–21.42	10.89 ± 2.43	11.13 ± 2.47	10.97 ± 2.27	10.63 ± 2.51	*p* = 0.33	*p* = 0.14
AA (% of fats)	0.27–0.73	0.47 ± 0.08	0.48 ± 0.09	0.47 ± 0.09	0.46 ± 0.07	*p* = 0.07	*p* = 0.01
ω-3 (% of fats)	0.68–4.40	1.21 ± 0.45	1.07 ± 0.25	1.22 ± 0.49	1.31 ± 0.51	*p* < 0.01	*p* < 0.01
ALA (% of fats)	0.27–1.15	0.54 ± 0.17	0.49 ± 0.14	0.54 ± 0.17	0.57 ± 0.18	*p* < 0.01	*p* < 0.01
EPA (% of fats)	0.01–0.38	0.06 ± 0.04	0.04 ± 0.02	0.06 ± 0.05	0.06 ± 0.05	*p* < 0.01	*p* < 0.01
DHA (% of fats)	0.09–2.30	0.29 ± 0.22	0.25 ± 0.12	0.30 ± 0.24	0.33 ± 0.26	*p* = 0.03	*p* = 0.01
DPA (% of fats)	0.03–0.59	0.12 ± 0.06	0.11 ± 0.03	0.13 ± 0.06	0.13 ± 0.07	*p* = 0.02	*p* = 0.02
ω-6/ω-3	2.50–28.28	11.17 ± 3.70	12.40 ± 3.79	11.13 ± 3.57	10.13 ± 3.39	*p* < 0.01	*p* < 0.01
LA/ALA	5.95–54.09	21.87 ± 7.40	24.05 ± 7.21	21.90 ± 7.63	19.93 ± 6.92	*p* < 0.01	*p* < 0.01
AA/LA	0.02–0.09	0.04 ± 0.01	0.05 ± 0.01	0.04 ± 0.01	0.04 ± 0.01	*p* = 0.71	*p* = 0.67
DHA/ALA	0.12–4.56	0.57 ± 0.38	0.54 ± 0.30	0.56 ± 0.31	0.59 ± 0.46	*p* = 0.63	*p* = 0.35
DHA/AA	0.23–5.25	0.64 ± 0.48	0.52 ± 0.23	0.66 ± 0.61	0.72 ± 0.53	*p* = 0.01	*p* < 0.01
(EPA + DHA)/ALA	0.14–5.00	0.67 ± 0.43	0.64 ± 0.35	0.67 ± 0.37	0.70 ± 0.52	*p* = 0.56	*p* = 0.29
(EPA + DHA)/AA	0.29–6.21	0.76 ± 0.57	0.61 ± 0.27	0.79 ± 0.74	0.86 ± 0.62	*p* < 0.01	*p* < 0.01

*^a^Adjusted for maternal energy intake/maternal pre-pregnancy weight (kcal/day/kg).*

*ω-3, omega-3; ω-6, omega-6; AA, arachidonic acid; ALA, α-linolenic acid; DHA, decosahexaenoic acid; DPA, docosapentaenoic acid; CA, capric acid; CPA, caprylic acid; EA, euricic acid; EPA, eicosapentaenoic acid; FA, fatty acid; LA, linoleic acid; LAU, lauric acid; MA, myristic acid; MDS, Mediterranean Diet Score; MUFA, monounsaturated fatty acid; OA, oleic acid; PA, palmitic acid; PUFA, polyunsaturated fatty acid; SA, stearic acid; SD, standard deviation; SFA, saturated fatty acid. MEDIDIET study, 2012–2017.*

Conversely, high adherence to the Mediterranean diet was associated with a significantly higher content of MUFA in human milk (43.27 ± 4.27% of fats for MDS = 0–3 points; 43.27 ± 4.47% of fats for MDS = 4 points; 45.24 ± 5.22% of fats for MDS = 5–8 points; *p* ANOVA and *p* for trend < 0.01). Both the oleic acid (OA) and the erucic acid (EA), the major types of MUFA, significantly increased according to the adherence to the Mediterranean diet.

The content of PUFA in human milk was not associated with the adherence to the Mediterranean diet (*p* ANOVA = 0.62 and *p* for trend = 0.35). However, considering the major types of PUFA, a significantly lower content of arachidonic acid (AA) in human milk was observed among mothers with high adherence to the Mediterranean diet (0.48 ± 0.09% of fats for MDS = 0–3 points; 0.47 ± 0.09% of fats for MDS = 4 points; 0.46 ± 0.07% of fats for MDS = 5–8 points; *p* for trend = 0.01). Conversely, a significantly higher content of ω-3 FA in human milk was observed among mothers highly adherent to the Mediterranean diet (1.07 ± 0.25% of fats for MDS = 0–3 points, 1.22 ± 0.49% of fats for MDS = 4 points, and 1.31 ± 0.51% of fats for MDS = 5–8 points; *p* ANOVA and *p* for trend < 0.01). Likewise, significantly higher content of the major types of ω-3 FA in human milk (i.e., α-linolenic acid – ALA, eicosapentaenoic acid – EPA, docosahexaenoic acid – DHA, docosapentaenoic acid – DPA) was observed among mothers with high adherence to the Mediterranean diet. In addition, high adherence to the Mediterranean diet was associated with a significantly lower ratio between ω-6 and ω-3 FA (*p* ANOVA and *p* for trend < 0.01) and the ratio between LA and ALA (*p* ANOVA and *p* for trend < 0.01) in human milk. Conversely, high adherence to the Mediterranean diet was associated with a higher DHA/AA (*p* ANOVA = 0.01; *p* for trend < 0.01) and (EPA + DHA)/AA (*p* ANOVA and *p* for trend < 0.01) in human milk. No clear association was observed for the others FA considered.

### Content of Fatty Acids in Human Milk According to the Adherence of Breastfeeding Mothers to Each Component of Mediterranean Diet Score

Table 4 reports the content of SFA in human milk according to the adherence of breastfeeding mothers to each component of MDS. For components more frequently consumed in the Mediterranean diet, one point (i.e., adherence) corresponded to an individual intake greater or equal to the median intake of vegetables, fruit, cereals, legumes, MUFA/SFA, and fish, respectively; for components less frequently consumed in the Mediterranean diet, one point corresponded to an individual intake less than the median intake of dairy products and meat, respectively; for alcohol component, one point corresponded to an individual alcohol intake ranging from 5 to less than 25 g of ethanol/day. Mothers who adhered to the vegetable component showed human milk with a significantly lower content of SFA (41.16 ± 5.29% of fats) than the human milk of non-adherent mothers (42.64 ± 4.41% of fats; *p* = 0.01). Likewise, mothers who were adherent to MUFA/SFA (41.07 ± 5.15 vs. 42.74 ± 4.52% of fats; *p* < 0.01) and dairy products (41.24 ± 4.71 vs. 42.58 ± 5.01; *p* = 0.02) components showed lower content of SFA in human milk. A similar pattern was observed for PA and SA.

**TABLE 4:** Mean and standard deviation contents of major saturated fatty acids in human milk (expressed as% of fats) according to the adherence of breastfeeding mothers to single components of the Mediterranean Diet Score.

MDS component	FA in human milk (% of fats)						
	SFA	CPA	CA	LAU	MA	PA	SA
	Mean ± SD	Mean ± SD	Mean ± SD	Mean ± SD	Mean ± SD	Mean ± SD	Mean ± SD
**Vegetables^a^**							
0 point	42.64 ± 4.41	0.24 ± 0.07	1.47 ± 0.43	5.20 ± 1.88	5.50 ± 1.55	23.02 ± 2.29	5.92 ± 1.13
1 point	41.16 ± 5.29	0.25 ± 0.08	1.53 ± 0.41	5.16 ± 1.97	5.39 ± 1.77	21.84 ± 2.38	5.68 ± 0.91
*p*-value (*t*-test)^b^	*p* = 0.01	*p* = 0.19	*p* = 0.22	*p* = 0.85	*p* = 0.60	*p* < 0.01	*p* = 0.06
**Fruit^a^**							
0 point	41.41 ± 4.92	0.24 ± 0.07	1.44 ± 0.43	4.99 ± 1.91	5.26 ± 1.62	22.32 ± 2.38	5.85 ± 1.00
1 point	42.37 ± 4.86	0.25 ± 0.07	1.54 ± 0.41	5.34 ± 1.93	5.61 ± 1.67	22.56 ± 2.43	5.76 ± 1.06
*p*-value (*t*-test)^b^	*p* = 0.10	*p* = 0.20	*p* = 0.04	*p* = 0.13	*p* = 0.08	*p* = 0.40	*p* = 0.47
**Cereals^a^**							
0 point	41.89 ± 5.22	0.25 ± 0.08	1.49 ± 0.44	5.19 ± 2.07	5.45 ± 1.75	22.45 ± 2.48	5.76 ± 0.96
1 point	41.96 ± 4.58	0.24 ± 0.07	1.50 ± 0.40	5.17 ± 1.77	5.44 ± 1.55	22.46 ± 2.33	5.85 ± 1.11
*p*-value (*t*-test)^b^	*p* = 0.91	*p* = 0.60	*p* = 0.90	*p* = 0.92	*p* = 0.95	*p* = 0.98	*p* = 0.46
**Legumes^a^**							
0 point	41.90 ± 4.85	0.24 ± 0.07	1.46 ± 0.44	5.08 ± 1.85	5.37 ± 1.64	22.64 ± 2.29	5.81 ± 1.00
1 point	41.95 ± 4.97	0.25 ± 0.07	1.53 ± 0.41	5.28 ± 2.00	5.52 ± 1.67	22.26 ± 2.50	5.80 ± 1.08
*p*-value (*t*-test)^b^	*p* = 0.93	*p* = 0.07	*p* = 0.18	*p* = 0.40	*p* = 0.46	*p* = 0.19	*p* = 0.89
**MUFA/SFA^a^**							
0 point	42.74 ± 4.52	0.24 ± 0.08	1.49 ± 0.45	5.22 ± 1.94	5.58 ± 1.66	22.97 ± 2.14	5.94 ± 1.01
1 point	41.07 ± 5.15	0.25 ± 0.07	1.51 ± 0.39	5.14 ± 1.91	5.32 ± 1.65	21.91 ± 2.54	5.67 ± 1.05
*p*-value (*t*-test)^b^	*p* < 0.01	*p* = 0.61	*p* = 0.71	*p* = 0.73	*p* = 0.19	*p* < 0.01	*p* = 0.03
**Fish^a^**							
0 point	42.38 ± 4.34	0.24 ± 0.07	1.49 ± 0.38	5.23 ± 1.83	5.49 ± 1.51	22.74 ± 2.24	5.87 ± 1.02
1 point	41.52 ± 5.35	0.25 ± 0.08	1.50 ± 0.45	5.13 ± 2.01	5.41 ± 1.78	21.19 ± 2.52	5.74 ± 1.05
*p*-value (*t*-test)^b^	*p* = 0.14	*p* = 0.58	*p* = 0.86	*p* = 0.67	*p* = 0.66	*p* = 0.06	*p* = 0.29
**Dairy products^c^**							
0 point	42.58 ± 5.01	0.25 ± 0.08	1.53 ± 0.45	5.25 ± 1.94	5.63 ± 1.73	22.70 ± 2.35	5.88 ± 1.03
1 point	41.24 ± 4.71	0.24 ± 0.07	1.47 ± 0.39	5.10 ± 1.91	5.26 ± 1.55	22.19 ± 2.43	5.72 ± 1.04
*p*-value (*t*-test)^b^	*p* = 0.02	*p* = 0.16	*p* = 0.26	*p* = 0.53	*p* = 0.06	*p* = 0.07	*p* = 0.19
**Meat^c^**							
0 point	41.96 ± 4.77	0.24 ± 0.07	1.48 ± 0.45	5.12 ± 2.07	5.37 ± 1.73	22.57 ± 2.28	5.89 ± 1.05
1 point	41.89 ± 5.05	0.25 ± 0.07	1.51 ± 0.39	5.24 ± 1.77	5.53 ± 1.58	22.33 ± 2.52	5.72 ± 1.02
*p*-value (*t*-test)^b^	*p* = 0.90	*p* = 0.61	*p* = 0.58	*p* = 0.57	*p* = 0.43	*p* = 0.40	*p* = 0.16
**Alcohol^d^**							
0 point	41.96 ± 4.93	0.24 ± 0.07	1.50 ± 0.42	5.19 ± 1.92	5.46 ± 1.66	22.46 ± 2.44	5.81 ± 1.04
1 point	41.26 ± 4.50	0.23 ± 0.08	1.47 ± 0.47	5.05 ± 1.97	5.15 ± 1.52	22.36 ± 1.61	5.77 ± 0.97
*p*-value (*t*-test)^b^	*p* = 0.60	*p* = 0.55	*p* = 0.80	*p* = 0.79	*p* = 0.48	*p* = 0.89	*p* = 0.89

*^a^1 point is assigned to an individual intake greater or equal to the median intake reported in the Table 1; ^b^Adjusted for maternal energy intake/maternal pre-pregnancy weight (kcal/day/kg); ^c^1 point is assigned to an individual intake less than the median intake reported in the Table 1; ^d^1 point is assigned to an individual intake ranging from 5 to less than 25 g of ethanol/day.*

*CA, capric acid; CPA, caprylic acid; FA, fatty acid; LAU, lauric acid; MA, myristic acid; MDS, Mediterranean Diet Score; PA, palmitic acid; SA, stearic acid; SD, standard deviation; SFA, saturated fatty acid. MEDIDIET study, 2012–2017.*

Table 5 reports the content of MUFA in human milk according to the adherence of breastfeeding mothers to each component of MDS. A significantly higher content of MUFA was observed for mothers who adhered to the vegetables (44.73 ± 5.34 vs. 43.41 ± 4.14% of fats; *p* = 0.02), MUFA/SFA (44.90 ± 5.03 vs. 43.23 ± 4.42% of fats; *p* < 0.01), and dairy products (44.87 ± 4.47 vs. 43.26 ± 4.97% of fats; *p* < 0.01) components. The adherence of such components resulted in human milk with higher contents of OA and EA.

**TABLE 5:** Mean and standard deviation contents of major monounsaturated fatty acids in human milk (expressed as% of fats) according to the adherence of breastfeeding mothers to single components of the Mediterranean Diet Score.

MDS component	FA in human milk (% of fats)		
	MUFA	OA	EA
	Mean ± SD	Mean ± SD	Mean ± SD
**Vegetables^a^**			
0 point	43.41 ± 4.14	38.65 ± 3.84	0.07 ± 0.02
1 point	44.73 ± 5.34	39.98 ± 5.15	0.08 ± 0.02
*p*-value (*t*-test)^b^	*p* = 0.02	*p* = 0.01	*p* < 0.01
**Fruit^a^**			
0 point	44.49 ± 4.86	39.76 ± 4.69	0.07 ± 0.02
1 point	43.67 ± 4.72	38.89 ± 4.42	0.08 ± 0.02
*p*-value (*t*-test)^b^	*p* = 0.15	*p* = 0.11	*p* = 0.80
**Cereals^a^**			
0 point	43.90 ± 5.02	39.14 ± 4.75	0.08 ± 0.02
1 point	44.19 ± 4.57	39.44 ± 4.37	0.07 ± 0.02
*p*-value (*t*-test)^b^	*p* = 0.62	*p* = 0.58	*p* = 0.08
**Legumes^a^**			
0 point	44.06 ± 4.81	39.29 ± 4.54	0.08 ± 0.02
1 point	44.03 ± 4.79	39.29 ± 4.59	0.08 ± 0.02
*p*-value (*t*-test)^b^	*p* = 0.95	*p* = 0.99	*p* = 0.88
**MUFA/SFA^a^**			
0 point	43.23 ± 4.42	38.47 ± 4.11	0.07 ± 0.02
1 point	44.90 ± 5.03	40.14 ± 4.85	0.08 ± 0.02
*p*-value (*t*-test)^b^	*p* < 0.01	*p* < 0.01	*p* < 0.01
**Fish^a^**			
0 point	43.73 ± 4.15	38.95 ± 3.95	0.07 ± 0.02
1 point	44.33 ± 5.31	39.59 ± 5.05	0.08 ± 0.02
*p*-value (*t*-test)^b^	*p* = 0.29	*p* = 0.24	*p* = 0.34
**Dairy products^c^**			
0 point	43.26 ± 4.97	38.55 ± 4.67	0.07 ± 0.02
1 point	44.87 ± 4.47	40.07 ± 4.32	0.08 ± 0.02
*p*-value (*t*-test)^b^	*p* < 0.01	*p* < 0.01	*p* = 0.05
**Meat^c^**			
0 point	43.97 ± 4.59	39.18 ± 4.42	0.08 ± 0.02
1 point	44.13 ± 5.01	39.40 ± 4.71	0.07 ± 0.02
*p*-value (*t*-test)^b^	*p* = 0.78	*p* = 0.69	*p* = 0.76
**Alcohol^d^**			
0 point	44.00 ± 4.80	39.24 ± 4.58	0.08 ± 0.02
1 point	44.97 ± 4.72	40.24 ± 4.23	0.08 ± 0.01
*p*-value (*t*-test)^b^	*p* = 0.46	*p* = 0.42	*p* = 0.74

*^a^1 point is assigned to an individual intake greater or equal to the median intake reported in the Table 1; ^b^Adjusted for maternal energy intake/maternal pre-pregnancy weight (kcal/day/kg); ^c^1 point is assigned to an individual intake less than the median intake reported in the Table 1; ^d^1 point is assigned to an individual intake ranging from 5 to less than 25 g of ethanol/day.*

*EA, euricic acid; FA, fatty acid; MDS, Mediterranean Diet Score; MUFA, monounsaturated fatty acid; OA, oleic acid; SD, standard deviation; SFA, saturated fatty acid. MEDIDIET study, 2012–2017.*

Table 6 reports the content of PUFA in human milk according to the adherence of breastfeeding mothers to each component of MDS. A significantly higher content of ω-3 FA was observed for mothers who adhered to the vegetables (1.30 ± 0.48 vs. 1.12 ± 0.39% of fats *p* < 0.01), MUFA/SFA (1.30 ± 0.56 vs 1.12 ± 0.27; *p* < 0.01), and fish (1.29 ± 0.50 vs. 1.12 ± 0.36; *p* < 0.01). Similarly, the adherence of breastfeeding mothers to vegetables, MUFA/SFA, and fish components was significantly associated with human milk with higher contents of ALA, EPA, DHA, and DPA.

**TABLE 6:** Mean and standard deviation contents of major polyunsaturated fatty acids in human milk (expressed as% of fats) according to the adherence of breastfeeding mothers to single components of the Mediterranean Diet Score.

MDS component	FA in human milk (% of fats)								
	PUFA	ω-6	LA	AA	ω-3	ALA	EPA	DHA	DPA
	Mean ± SD	Mean ± SD	Mean ± SD	Mean ± SD	Mean ± SD	Mean ± SD	Mean ± SD	Mean ± SD	Mean ± SD
**Vegetables^a^**									
0 point	13.52 ± 2.40	12.40 ± 2.34	10.90 ± 2.27	0.47 ± 0.08	1.12 ± 0.39	0.50 ± 0.15	0.05 ± 0.04	0.27 ± 0.18	0.11 ± 0.05
1 point	13.67 ± 2.66	12.37 ± 2.69	10.88 ± 2.61	0.46 ± 0.08	1.30 ± 0.48	0.57 ± 0.17	0.06 ± 0.05	0.32 ± 0.25	0.13 ± 0.06
*p*-value (*t*-test)^b^	*p* = 0.61	*p* = 0.93	*p* = 0.94	*p* = 0.26	*p* < 0.01	*p* < 0.01	*p* = 0.06	*p* = 0.04	*p* < 0.01
**Fruit^a^**									
0 point	13.66 ± 2.43	12.48 ± 2.41	10.99 ± 2.34	0.47 ± 0.09	1.18 ± 0.41	0.53 ± 0.15	0.05 ± 0.04	0.29 ± 0.21	0.12 ± 0.05
1 point	13.53 ± 2.61	12.30 ± 2.59	10.81 ± 2.52	0.47 ± 0.08	1.23 ± 0.47	0.54 ± 0.18	0.06 ± 0.05	0.30 ± 0.23	0.12 ± 0.06
*p*-value (*t*-test)^b^	*p* = 0.65	*p* = 0.55	*p* = 0.53	*p* = 0.43	*p* = 0.39	*p* = 0.50	*p* = 0.49	*p* = 0.62	*p* = 0.49
**Cereals^a^**									
0 point	13.76 ± 2.32	12.51 ± 2.29	11.02 ± 2.23	0.47 ± 0.08	1.26 ± 0.51	0.55 ± 0.19	0.06 ± 0.05	0.32 ± 0.27	0.12 ± 0.06
1 point	13.42 ± 2.70	12.27 ± 2.70	10.77 ± 2.62	0.47 ± 0.09	1.15 ± 0.36	0.52 ± 0.14	0.05 ± 0.04	0.27 ± 0.15	0.12 ± 0.05
*p*-value (*t*-test)^b^	*p* = 0.25	*p* = 0.43	*p* = 0.39	*p* = 0.74	*p* = 0.05	*p* = 0.09	*p* = 0.19	*p* = 0.06	*p* = 0.23
**Legumes^a^**									
0 point	13.61 ± 2.51	12.42 ± 2.48	10.93 ± 2.42	0.47 ± 0.09	1.19 ± 0.44	0.53 ± 0.16	0.06 ± 0.05	0.29 ± 0.22	0.12 ± 0.05
1 point	13.57 ± 2.55	12.35 ± 2.55	10.85 ± 2.45	0.47 ± 0.08	1.23 ± 0.45	0.54 ± 0.17	0.06 ± 0.04	0.30 ± 0.22	0.12 ± 0.06
*p*-value (*t*-test)^b^	*p* = 0.90	*p* = 0.80	*p* = 0.79	*p* = 0.45	*p* = 0.44	*p* = 0.48	*p* = 0.93	*p* = 0.86	*p* = 0.83
**MUFA/SFA^a^**									
0 point	13.59 ± 2.65	12.47 ± 2.65	10.97 ± 2.58	0.48 ± 0.09	1.12 ± 0.27	0.51 ± 0.15	0.05 ± 0.03	0.26 ± 0.12	0.11 ± 0.04
1 point	13.59 ± 2.40	12.30 ± 2.36	10.81 ± 2.28	0.46 ± 0.08	1.30 ± 0.56	0.57 ± 0.18	0.06 ± 0.06	0.33 ± 0.29	0.13 ± 0.07
*p*-value (*t*-test)^b^	*p* = 0.98	*p* = 0.57	*p* = 0.60	*p* = 0.24	*p* < 0.01	*p* < 0.01	*p* < 0.01	*p* = 0.01	*p* = 0.01
**Fish^a^**									
0 point	13.45 ± 2.40	12.34 ± 2.41	10.83 ± 2.34	0.47 ± 0.09	1.12 ± 0.36	0.51 ± 0.16	0.05 ± 0.04	0.25 ± 0.15	0.11 ± 0.05
1 point	13.72 ± 2.63	12.43 ± 2.60	10.94 ± 2.52	0.47 ± 0.08	1.29 ± 0.50	0.55 ± 0.17	0.06 ± 0.05	0.33 ± 0.26	0.13 ± 0.06
*p*-value (*t*-test)^b^	*p* = 0.39	*p* = 0.75	*p* = 0.71	*p* = 0.98	*p* < 0.01	*p* = 0.04	*p* < 0.01	*p* < 0.01	*p* = 0.03
**Dairy products^c^**									
0 point	13.71 ± 2.76	12.50 ± 2.77	10.99 ± 2.70	0.47 ± 0.09	1.20 ± 0.42	0.52 ± 0.16	0.06 ± 0.04	0.30 ± 0.21	0.12 ± 0.06
1 point	13.47 ± 2.25	12.26 ± 2.19	10.79 ± 2.12	0.46 ± 0.08	1.21 ± 0.47	0.55 ± 0.17	0.05 ± 0.05	0.29 ± 0.23	0.12 ± 0.05
*p*-value (*t*-test)^b^	*p* = 0.44	*p* = 0.42	*p* = 0.49	*p* = 0.39	*p* = 0.87	*p* = 0.16	*p* = 0.61	*p* = 0.83	*p* = 0.32
**Meat^c^**									
0 point	13.64 ± 2.36	12.42 ± 2.37	10.89 ± 2.30	0.48 ± 0.09	1.22 ± 0.48	0.54 ± 0.17	0.06 ± 0.05	0.29 ± 0.24	0.12 ± 0.06
1 point	13.54 ± 2.68	12.35 ± 2.64	10.89 ± 2.57	0.45 ± 0.08	1.19 ± 0.41	0.53 ± 0.16	0.05 ± 0.04	0.29 ± 0.20	0.12 ± 0.05
*p*-value (*t*-test)^b^	*p* = 0.76	*p* = 0.82	*p* = 0.99	*p* < 0.01	*p* = 0.65	*p* = 0.58	*p* = 0.73	*p* = 0.99	*p* = 0.32
**Alcohol^d^**									
0 point	13.60 ± 2.55	12.39 ± 2.53	10.90 ± 2.46	0.47 ± 0.08	1.21 ± 0.45	0.54 ± 0.17	0.06 ± 0.05	0.29 ± 0.23	0.12 ± 0.06
1 point	13.38 ± 1.96	12.31 ± 2.01	10.68 ± 1.89	0.51 ± 0.10	1.07 ± 0.16	0.48 ± 0.10	0.05 ± 0.01	0.27 ± 0.08	0.11 ± 0.02
*p*-value (*t*-test)^b^	*p* = 0.75	*p* = 0.91	*p* = 0.74	*p* = 0.07	*p* = 0.25	*p* = 0.17	*p* = 0.41	*p* = 0.63	*p* = 0.49

*^a^1 point is assigned to an individual intake greater or equal to the median intake reported in the Table 1; ^b^Adjusted for maternal energy intake/maternal pre-pregnancy weight (kcal/day/kg); ^c^1 point is assigned to an individual intake less than the median intake reported in the Table 1; ^d^1 point is assigned to an individual intake ranging from 5 to less than 25 g of ethanol/day.*

*ω-3, omega-3; ω-6, omega-6; AA, arachidonic acid; ALA, α-linolenic acid; DHA, decosahexaenoic acid; DPA, docosapentaenoic acid; EPA, eicosapentaenoic acid; LA, linoleic acid; MDS, Mediterranean Diet Score; MUFA, monounsaturated fatty acid; PUFA, polyunsaturated fatty acid; SD, standard deviation; SFA, saturated fatty acid. MEDIDIET study, 2012–2017.*

## Discussion

We assessed the association between the adherence of breastfeeding mothers to the Mediterranean diet and the FA content of their human milk using data from the Italian MEDIDIET study. High adherence to the Mediterranean diet was associated with lower content of SFA (including PA and SA), ω-6/ω-3, and LA/ALA, and higher content of MUFA (including OA and EA), ω-3 FA (including ALA, EPA, DHA, and DPA), and DHA/AA and (EPA + DHA)/AA) in human milk. These associations were mainly driven by the adherence to the vegetables, MUFA/SFA, fish, and dairy products components of the Mediterranean diet.

Previous studies investigated the relationship between maternal intakes of single foods or nutrients and the FA composition in human milk. A longitudinal Finnish study (25) reported a weak positive correlation (*r* = 0.21) between maternal intake of high-fat dairy products and the content of SFA in human milk, collected at 3 months of lactation. Likewise, we observed a lower content of SFA in human milk for mothers with high adherence to the Mediterranean diet, which is characterized by a moderate to low consumption of dairy products. Accordingly, two studies (26, 27) found positive correlations between maternal intake of SFA and the content of SFA in human milk (*r* = 0.60 and *r* = 0.215, respectively). The lower content of SFA in human milk observed among mothers with high adherence to the Mediterranean diet could not provide optimal food for the infant’s needs (2, 28–31). However, the mean content of SFA in human milk among mothers who highly adhered to the Mediterranean diet (40.92 ± 5.22% of fats) was in agreement with the typical human milk composition of FA, which contains approximately 35–45% of SFA, 45–50% of MUFA, and 15% of PUFA (32, 33).

The Italian cross-sectional study by Scopesi et al. (26) reported a positive correlation of 0.63 between maternal intake of MUFA and the content of MUFA in human milk (collected during the 1st month of lactation) which is in agreement with the higher content of MUFA in human milk observed in this study for mothers who were adherent to the Mediterranean diet. The higher content of MUFA in human milk for these mothers likely derives from the consumption of olive oil, the major fat source of the Mediterranean diet, which is rich in MUFA, especially in the form of OA (34).

A cross-sectional study conducted in Denmark (35) reported a higher content of ω-3 FA (including EPA, DHA, and DPA) in human milk (collected at 4 months postpartum) for mothers who had a high intake of fish, as compared to mothers who did not consume it. A longitudinal Greek study (36) reported positive correlations between maternal PUFA intake and the contents of ω-3 FA (*r* = 0.26) and DHA (*r* = 0.27) in human milk, collected in the 1st month of lactation. Likewise, the cross-sectional study of Kim et al. (27) reported a weak positive correlation (*r* = 0.211) between maternal intake of PUFA and the content of ω-3 FA in human milk (collected from 1 to 11 months postpartum) for South Korean mothers. The cross-sectional study by Olafsdottir et al. (37) reported a positive correlation of 0.43 between maternal PUFA intake and the content of ALA in human milk (collected from 2 to 4 months postpartum) for Icelandic mothers. We observed a higher content of ω-3 FA and the major types of ω-3 FA in human milk for mothers with high adherence to the Mediterranean diet. The moderate consumption of fish jointly with the high consumption of vegetables (various of them are relatively rich in ω-3 FA) results in an appreciable intake of ω-3 FA for populations belonging to the Mediterranean area (34).

Three Chinese studies assessed the association between *a posteriori* dietary patterns of breastfeeding mothers and the composition of human milk (6–8). Dietary patterns were derived through principal component analysis based on food group intakes of mothers. Among the dietary patterns identified in the cross-sectional study by Hu et al. (6), the “high-in-plant-foods” dietary pattern was negatively associated with total fats content in human milk, collected within 2 months postpartum. We observed a lower content of SFA, a higher content of MUFA, and a stationary content of PUFA in human milk for high adherence to the Mediterranean diet. In the cross-sectional study by Tian et al. (7), the dietary pattern characterized by “dairy and soybean products, and nuts” showed higher content of SFA in human milk; the dietary pattern characterized by “meats, mushrooms and algae, and marine products” and the dietary pattern characterized by “vegetables and fruit” showed higher content of PUFA and ω-6 FA in human milk (collected from 22 days to 6 months postpartum). In our study, neither the adherence to the Mediterranean diet nor the adherence to vegetables, fruit, and fish components was associated with the contents of PUFA and ω-6 FA in human milk. No dietary pattern identified in the cross-sectional study of Huang et al. was associated with the FA profile in human milk, collected in the postpartum period from 7 days to 6 months and beyond (8). In our previous analysis of the same data, we identified five *a posteriori* dietary patterns based on nutrient intakes of breastfeeding mothers (5). The nutritional profile of three out of five dietary patterns, named “Proteins and FA with legs,” “FA with fins,” and “FA with leaves,” was consistent with the Mediterranean diet and therefore they showed similar associations with the FA content in human milk. In particular, the “Proteins and FA with legs” dietary pattern, characterized by high maternal intake of animal proteins (including dairy products), was weakly positively correlated (*r* = 0.12) with the content of SFA and weakly negatively correlated (*r* = −0.20) with the content of MUFA in human milk. The “FA with fins” dietary pattern, characterized by high maternal intake of fish, was weakly positively correlated (*r* = 0.23) with the content of ω-3 FA (including ALA, EPA, DHA, and DPA) in human milk. The “FA with leaves,” dietary pattern characterized by a high intake of vegetables, was weakly positively correlated (*r* = 0.17) with the content of MUFA and weakly negatively correlated (*r* = −0.19) with the content of SFA in human milk.

The FA in human milk derives from the endogenous synthesis in the mammary gland and uptake from maternal plasma. In both cases, the content of FA in human milk may be influenced by maternal nutrition (2, 38, 39). Furthermore, the role of FA in the infant diet is gaining interest because FA is involved in several growth processes, especially in visual and neural functions (28, 40). In particular, DHA is selectively enriched in a few specific membrane lipids, which include the glycerophospholipids of the retina and brain gray matter (29). High concentrations of DHA are present in phosphatidylserine and the ethanolamine phosphoacylglycerols of gray matter and the outer segments of rod and cone photoreceptors in the retina (41). Considerable evidence showed that poor maternal DHA status increased the risk of inadequate transferring of DHA in human milk and consequently a delayed or reduced neural and visual system development of the infant (30, 42–44).

### Strengths and Limitations

We observed some maternal dietary behaviors diverging from the typical Mediterranean diet such as an overall low intake of legumes, an overall quite high intake of dairy products and meat, and a low intake of vegetables for mothers from Southern Italy (Supplementary Table 1). This could be partially explained by the results of a recent internet survey describing the nutritional behavior of the Italian population and their compliance with the Mediterranean pyramid recommendations. The authors reported “a rather low adherence” of participants to some Mediterranean pyramid recommendations, especially by females and in the South (45). In addition, we observed that the human milk of mothers in the South had a lower content of ω-6 and ALA (Supplementary Table 2), likely due to these changing dietary habits. Nevertheless, we generally did not find other modified associations between the adherence to the Mediterranean diet and the content of FA in human milk across the strata of geographical area (Supplementary Table 2).

Some studies showed differences in the FA profile of human milk according to maternal pre-pregnancy weight and/or BMI (46, 47). However, no difference emerged in the distributions of maternal pre-pregnancy weight and BMI according to the adherence to the MDS, excluding possible confounding effects of these variables (Supplementary Table 3). Nevertheless, mothers highly adherent to the Mediterranean diet tended to be older than those with low or medium adherence, not excluding a possible residual confounding due to age (Supplementary Table 3). In addition, the cross-sectional design of this study did not allow to establish a causal relationship between the Mediterranean diet and the content of FA in human milk. Nevertheless, it is unlikely that differences in the contents of FA in human milk could have modified the maternal adherence to the Mediterranean diet. However, it could not be ruled out that the existence of external factors (e.g., socioeconomic status or healthy lifestyles) trigger the adherence to the Mediterranean diet and the composition of human milk in parallel and causatively influence the content of FA. Dietary information of breastfeeding mothers was self-reported and possibly biased from incomplete recall. However, it was collected by trained interviewers using structured FFQ which has been validated (18) and tested for the reproducibility (19, 20) in the Italian adult population, minimizing possible recall bias. Although we used a validated and reproducible FFQ, the maternal nutrient intakes could be underestimated. Nevertheless, we previously compared maternal nutrient intakes of mothers participating in the MEDIDIET study with those of mothers from other developed countries in a reviewing framework. The energy intake of mothers participating in the MEDIDIET study was lower than the average energy requirement (i.e., 2,300 kcal/day) recommended during the lactation period, but it was in agreement with those of the majority of studies included in the review (48).

Possible systematic and random errors in the assessment of the content of FA in human milk were reduced by a standard protocol for the collection and analysis of human milk samples. In addition, we collected all human milk samples in a short window of time (i.e., 6 ± 1 weeks postpartum) and in the morning for all mothers participating in the MEDIDIET study, avoiding the variability due to different lactation stages or different times of the day. However, we did not collect human milk samples repeatedly over time making it difficult to make conclusions on the impact of maternal diet on the composition of human milk in a comprehensive way.

Another limitation of this study is the lack of information on follow-up of the infant growth. Thus, we can establish the relationship between maternal nutrition and human milk composition and only infer the relationship between human milk properties and infant outcomes using results from external studies.

The multicentric design of the MEDIDIET study allowed us to include mothers from different Italian regions strengthening the findings of this study. The inclusion of healthy breastfeeding mothers with healthy infants allowed transferring of these results to the general population. In addition, another strength includes the detailed set of FA in human milk provided in this analysis. Lastly, to the best of our knowledge, this is the first study aimed to evaluate the relationship between the Mediterranean diet and the content of FA in human milk using a formal score of adherence.

## Conclusion

High adherence to the Mediterranean diet was associated with human milk with lower content of SFA and higher contents of MUFA, ω-3 FA, and the major types of ω-3 FA, including DHA. The Mediterranean diet may contribute to human milk production with higher content of specific FA which is directly involved in the growth and development of neural and visual functions of the infant, as reported by previous studies. If the results of this study were confirmed by further analyses, possibly including infant outcomes, this would help in the development of dietary guidelines for breastfeeding mothers to promote good dietary practices – such as the Mediterranean diet – and their positive implications for infants.

## Data Availability Statement

The original contributions presented in this study are included in the article/Supplementary Material, further inquiries can be directed to the corresponding author.

## Ethics Statement

The studies involving human participants were reviewed and approved by the Ethics Committee of participating hospitals. The patients/participants provided their written informed consent to participate in this study.

## Medidiet Working Group Members

Ansaldi Giulia (Neonatal Care Unit of the University, City of Health and Science Hospital, Turin, Italy);

Amadio Patrizia (Ospedale Pediatrico Bambino Gesù, Rome, Italy);

Chester Elena (Neonatal Care Unit of the University, City of Health and Science Hospital, Turin, Italy);

Di Nicola Paola (Neonatal Care Unit of the University, City of Health and Science Hospital, Turin, Italy);

Monzali Francesca (Azienda Ospedaliera Universitaria Meyer, Florence, Italy);

Palumbo Giuseppina (“Casa Sollievo della Sofferenza” Foundation, San Giovanni Rotondo, Italy);

Roselli Elena (Azienda Ospedaliera Universitaria Meyer, Florence, Italy);

Siemens Louise (Ospedale Buccheri La Ferla, FBF, Palermo, Italy);

Sottemano Stefano (Neonatal Care Unit of the University, City of Health and Science Hospital, Turin, Italy);

Villani Antonio (“Casa Sollievo della Sofferenza” Foundation, San Giovanni Rotondo, Italy).

## Author Contributions

GM, MF, and AD designed the research idea. FB and MD performed the statistical analyses and wrote the manuscript. MF, FB, MD, CA, SE, EB, GM, and BS interpreted the results. GM directed data acquisition of the MEDIDIET study. PT, PQ, GS, CP, and IK managed data acquisition of the MEDIDIET study. All authors have read and approved the final version of the manuscript.

## Conflict of Interest

The statistical analyses were performed by the Department of Clinical Sciences and Community Health, Branch of Medical Statistics, Biometry and Epidemiology “G.A. Maccacaro”, Università degli Studi di Milano, Milan, Italy (MD, FB, and MF) without the involvement of the Sponsor. SE, EV, and BS were employees of Danone Nutricia Research, Utrecht, Netherlands. The remaining authors declare that the research was conducted in the absence of any commercial or financial relationships that could be construed as a potential conflict of interest.

## Publisher’s Note

All claims expressed in this article are solely those of the authors and do not necessarily represent those of their affiliated organizations, or those of the publisher, the editors and the reviewers. Any product that may be evaluated in this article, or claim that may be made by its manufacturer, is not guaranteed or endorsed by the publisher.
