# Supplementary material for: Adherence to Mediterranean Diet of Breastfeeding Mothers and Fatty Acids Composition of Their Human Milk: Results From the Italian MEDIDIET Study
Source: Front Nutr. 2022 Jun 2;9:891376. doi: 10.3389/fnut.2022.891376 (PMC9201754; doi:10.3389/fnut.2022.891376)
Supplement: Supplementary file 1 [file Table_1.DOCX]

Supplementary Material

**Supplementary table 1.** Distribution of components of the Mediterranean diet score according to geographical area. MEDIDIET study, 2012-2014.

| **Component** | **P10** | **P25** | **P50** | **P75** | **P90** | **Mean±SD** |
| --- | --- | --- | --- | --- | --- | --- |
|  |  |  |  |  |  |  |
| **North^a^** |  |  |  |  |  |  |
| Vegetables (servings/week) | 6.38 | 9.67 | 13.56 | 17.31 | 24.94 | 14.40±7.03 |
| Fruit (servings/week) | 5.75 | 10.38 | 14.88 | 17.50 | 22.25 | 14.49±6.89 |
| Cereals (servings/week) | 11.81 | 16.00 | 20.12 | 24.53 | 27.88 | 20.16±6.79 |
| Legumes (servings/week) | 0.19 | 0.64 | 1.22 | 2.00 | 2.94 | 1.44±1.20 |
| MUFA/SFA | 1.04 | 1.15 | 1.28 | 1.44 | 1.58 | 1.30±0.22 |
| Fish (servings/week) | 0.50 | 1.00 | 1.75 | 2.00 | 3.00 | 1.70±0.99 |
| Dairy products (servings/week) | 2.30 | 5.72 | 9.61 | 12.38 | 17.37 | 9.56±5.47 |
| Meat (servings/week) | 5.71 | 6.72 | 8.80 | 10.37 | 13.82 | 9.03±3.59 |
| Alcohol (grams/day) | 0.00 | 0.00 | 0.00 | 1.81 | 3.57 | 1.44±3.18 |
| **Central^b^** |  |  |  |  |  |  |
| Vegetables (servings/week) | 6.22 | 9.38 | 13.00 | 17.88 | 25.75 | 14.70±8.21 |
| Fruit (servings/week) | 4.60 | 9.00 | 14.00 | 18.50 | 27.60 | 15.23±10.53 |
| Cereals (servings/week) | 12.70 | 16.62 | 22.00 | 29.50 | 34.80 | 23.90±12.00 |
| Legumes (servings/week) | 0.00 | 0.65 | 1.38 | 2.25 | 3.75 | 1.72±1.68 |
| MUFA/SFA | 1.05 | 1.21 | 1.31 | 1.45 | 1.62 | 1.32±0.23 |
| Fish (servings/week) | 0.00 | 1.00 | 2.00 | 3.00 | 3.70 | 1.98±1.30 |
| Dairy products (servings/week) | 3.61 | 7.42 | 10.58 | 13.33 | 17.82 | 10.63±5.51 |
| Meat (servings/week) | 5.00 | 7.00 | 9.00 | 11.00 | 13.32 | 9.23±3.39 |
| Alcohol (grams/day) | 0.00 | 0.00 | 0.00 | 0.00 | 1.87 | 0.69±2.22 |
| **South^c^** |  |  |  |  |  |  |
| Vegetables (servings/week) | 4.00 | 5.38 | 8.00 | 11.00 | 14.20 | 8.64±4.76 |
| Fruit (servings/week) | 9.80 | 13.00 | 17.00 | 22.00 | 28.20 | 18.01±7.78 |
| Cereals (servings/week) | 15.95 | 19.12 | 24.75 | 31.50 | 39.57 | 26.24±9.86 |
| Legumes (servings/week) | 0.00 | 0.19 | 1.00 | 1.38 | 2.12 | 0.97±0.91 |
| MUFA/SFA | 0.97 | 1.07 | 1.20 | 1.31 | 1.52 | 1.21±0.21 |
| Fish (servings/week) | 0.50 | 1.00 | 2.00 | 2.00 | 3.00 | 1.63±0.99 |
| Dairy products (servings/week) | 5.80 | 9.38 | 12.50 | 16.75 | 19.95 | 12.67±5.65 |
| Meat (servings/week) | 4.39 | 6.12 | 7.25 | 9.12 | 10.63 | 7.52±2.49 |
| Alcohol (grams/day) | 0.00 | 0.00 | 0.00 | 0.00 | 1.87 | 0.71±1.86 |
|  |  |  |  |  |  |  |

^a^North includes breastfeeding mothers enrolled in Turin; ^b^Central includes breastfeeding mothers enrolled in Florence and Rome; ^c^South includes breastfeeding mothers enrolled in San Giovanni Rotondo and Palermo.

Abbreviations: MUFA, monounsaturated fatty acids; SD, standard deviation; SFA, saturated fatty acids.

**Supplementary table 2.** Means and standard deviations of selected fatty acids content (expresses as % of fats) in human milk according to the adherence of breastfeeding mothers to the Mediterranean diet score (expressed approximately in tertiles) in strata of geographical area. MEDIDIET study, 2012-2014.

| **FA in milk** | **Total** | |  | **MDS** | | | **p-value**  **(ANOVA)^a^** | **p-value**  **(trend)^a^** |
| --- | --- | --- | --- | --- | --- | --- | --- | --- |
|  |  |  |  | **0-3 points** | **4 points** | **5-8 points** |  |  |
|  | **min-max** | **mean±SD** |  | **mean±SD** | **mean±SD** | **mean±SD** |  |  |
|  |  |  |  |  |  |  |  |  |
| **North^b^** |  |  |  | **n=29 (30.21)** | **n=19 (19.79)** | **n=48 (50.00)** |  |  |
| SFA (% of fats) | 27.89-54.42 | 41.70±5.01 |  | 42.99±4.49 | 42.37±5.18 | 40.65±5.11 | p=0.11 | p=0.06 |
| MUFA (% of fats) | 31.65-60.82 | 44.28±5.13 |  | 43.08±4.16 | 43.75±5.01 | 45.22±5.61 | p=0.19 | p=0.07 |
| PUFA (% of fats) | 9.03-24.32 | 13.57±2.63 |  | 13.49±2.69 | 13.42±2.69 | 13.68±2.62 | p=0.92 | p=0.98 |
| ω-6 (% of fats) | 8.14-23.10 | 12.39±2.60 |  | 12.39±2.68 | 12.30±2.66 | 12.44±2.59 | p=0.98 | p=0.82 |
| LA (% of fats) | 6.41-21.42 | 10.92±2.51 |  | 10.88±2.61 | 10.83±2.55 | 10.99±2.49 | p=0.97 | p=0.91 |
| AA (% of fats) | 0.27-0.67 | 0.46±0.08 |  | 0.48±0.09 | 0.48±0.09 | 0.44±0.07 | p=0.06 | p=0.03 |
| ω-3 (% of fats) | 0.74-3.27 | 1.18±0.36 |  | 1.10±0.21 | 1.12±0.31 | 1.24±0.44 | p=0.18 | p=0.07 |
| ALA (% of fats) | 0.30-1.15 | 0.55±0.15 |  | 0.51±0.11 | 0.52±0.18 | 0.58±0.16 | p=0.11 | p=0.04 |
| EPA (% of fats) | 0.02-0.32 | 0.05±0.05 |  | 0.05±0.03 | 0.05±0.02 | 0.06±0.06 | p=0.49 | p=0.23 |
| DHA (% of fats) | 0.09-1.33 | 0.26±0.17 |  | 0.24±0.12 | 0.24±0.12 | 0.28±0.21 | p=0.51 | p=0.24 |
| DPA (% of fats) | 0.06-0.38 | 0.11±0.04 |  | 0.11±0.04 | 0.12±0.03 | 0.11±0.05 | p=0.78 | p=0.70 |
| ω-6/ω-3 | 3.40-19.98 | 11.16±3.27 |  | 11.59±3.21 | 11.53±3.15 | 10.76±3.36 | p=0.48 | p=0.16 |
| LA/ALA | 8.75-54.09 | 21.23±7.24 |  | 22.25±6.85 | 22.60±8.98 | 20.08±6.66 | p=0.29 | p=0.12 |
| AA/LA | 0.02-0.09 | 0.04±0.01 |  | 0.05±0.01 | 0.05±0.01 | 0.04±0.01 | p=0.08 | p=0.09 |
| DHA/ALA | 0.12-1.71 | 0.49±0.28 |  | 0.50±0.29 | 0.48±0.20 | 0.49±0.30 | p=0.98 | p=0.97 |
| DHA/AA | 0.24-2.22 | 0.57±0.34 |  | 0.50±0.23 | 0.51±0.25 | 0.63±0.42 | p=0.21 | p=0.08 |
| (EPA+DHA)/ALA | 0.14-2.11 | 0.59±0.34 |  | 0.59±0.34 | 0.58±0.24 | 0.59±0.37 | p=0.99 | p=0.93 |
| (EPA+DHA)/AA | 0.31-2.74 | 0.69±0.43 |  | 0.60±0.28 | 0.61±0.29 | 0.76±0.53 | p=0.20 | p=0.07 |
| **Central^c^** |  |  |  | **n=16 (23.88)** | **n=20 (29.85)** | **n=31 (46.27)** |  |  |
| SFA (% of fats) | 30.70-56.11 | 40.18±4.81 |  | 40.69±3.88 | 41.51±4.73 | 39.07±5.15 | p=0.19 | p=0.13 |
| MUFA (% of fats) | 33.55-54.43 | 45.26±4.70 |  | 44.61±4.09 | 43.76±4.73 | 46.56±4.74 | p=0.09 | p=0.08 |
| PUFA (% of fats) | 9.97-21.19 | 14.14±2.24 |  | 14.25±1.64 | 14.33±2.34 | 13.96±2.49 | p=0.83 | p=0.71 |
| ω-6 (% of fats) | 8.49-20.15 | 12.74±2.27 |  | 13.08±1.66 | 12.88±2.33 | 12.47±2.52 | p=0.65 | p=0.43 |
| LA (% of fats) | 6.85-18.24 | 11.23±2.20 |  | 11.56±1.60 | 11.34±2.21 | 10.99±2.48 | p=0.69 | p=0.46 |
| AA (% of fats) | 0.34-0.73 | 0.48±0.08 |  | 0.50±0.08 | 0.46±0.09 | 0.48±0.07 | p=0.35 | p=0.38 |
| ω-3 (% of fats) | 0.83-4.40 | 1.40±0.58 |  | 1.17±0.24 | 1.44±0.82 | 1.48±0.51 | p=0.20 | p=0.11 |
| ALA (% of fats) | 0.36-1.15 | 0.60±0.20 |  | 0.54±0.17 | 0.59±0.21 | 0.63±0.21 | p=0.30 | p=0.12 |
| EPA (% of fats) | 0.02-0.38 | 0.07±0.06 |  | 0.05±0.02 | 0.08±0.09 | 0.08±0.05 | p=0.14 | p=0.17 |
| DHA (% of fats) | 0.13-2.05 | 0.36±0.27 |  | 0.27±0.10 | 0.38±0.42 | 0.40±0.19 | p=0.30 | p=0.15 |
| DPA (% of fats) | 0.08-0.47 | 0.14±0.06 |  | 0.12±0.02 | 0.15±0.09 | 0.15±0.06 | p=0.25 | p=0.19 |
| ω-6/ω-3 | 2.59-20.33 | 10.23±3.76 |  | 11.63±2.78 | 10.72±4.81 | 9.19±3.21 | p=0.08 | p=0.03 |
| LA/ALA | 5.95-50.48 | 20.75±7.95 |  | 22.89±5.71 | 21.46±8.99 | 19.18±8.15 | p=0.29 | p=0.14 |
| AA/LA | 0.02-0.07 | 0.04±0.01 |  | 0.04±0.01 | 0.04±0.01 | 0.05±0.01 | p=0.58 | p=0.61 |
| DHA/ALA | 0.23-2.03 | 0.61±0.31 |  | 0.53±0.21 | 0.62±0.44 | 0.64±0.25 | p=0.48 | p=0.22 |
| DHA/AA | 0.25-5.25 | 0.79±0.68 |  | 0.57±0.27 | 0.88±1.10 | 0.85±0.42 | p=0.33 | p=0.24 |
| (EPA+DHA)/ALA | 0.25-2.40 | 0.72±0.37 |  | 0.61±0.24 | 0.75±0.53 | 0.76±0.29 | p=0.41 | p=0.22 |
| (EPA+DHA)/AA | 0.29-6.21 | 0.95±0.82 |  | 0.67±0.31 | 1.08±1.32 | 1.01±0.52 | p=0.28 | p=0.23 |
| **South^d^** | |  |  | **n=52 (43.70)** | **n=35 (29.41)** | **n=32 (26.89)** |  |  |
| SFA (% of fats) | 31.32-55.51 | 43.09±4.58 |  | 42.93±4.35 | 43.31±4.84 | 43.11±4.78 | p=0.93 | p=0.60 |
| MUFA (% of fats) | 31.12-53.05 | 43.17±4.42 |  | 42.97±4.38 | 42.73±4.06 | 43.98±4.87 | p=0.47 | p=0.52 |
| PUFA (% of fats) | 9.59-22.79 | 13.30±2.56 |  | 13.67±2.66 | 13.51±2.31 | 12.47±2.52 | p=0.09 | p=0.04 |
| ω-6 (% of fats) | 8.75-21.51 | 12.18±2.55 |  | 12.65±2.64 | 12.36±2.30 | 11.22±2.48 | p=0.04 | p=0.02 |
| LA (% of fats) | 7.51-19.85 | 10.67±2.49 |  | 11.13±2.63 | 10.84±2.18 | 9.75±2.41 | p=0.04 | p=0.02 |
| AA (% of fats) | 0.28-0.71 | 0.47±0.08 |  | 0.48±0.09 | 0.46±0.09 | 0.47±0.07 | p=0.59 | p=0.36 |
| ω-3 (% of fats) | 0.68-4.06 | 1.12±0.38 |  | 1.02±0.26 | 1.15±0.24 | 1.25±0.60 | p=0.03 | p=0.02 |
| ALA (% of fats) | 0.27-0.94 | 0.49±0.14 |  | 0.46±0.14 | 0.52±0.14 | 0.50±0.14 | p=0.10 | p=0.26 |
| EPA (% of fats) | 0.01-0.22 | 0.05±0.03 |  | 0.04±0.02 | 0.05±0.03 | 0.06±0.04 | p=0.04 | p=0.02 |
| DHA (% of fats) | 0.09-2.30 | 0.28±0.22 |  | 0.25±0.13 | 0.28±0.11 | 0.34±0.37 | p=0.18 | p=0.11 |
| DPA (% of fats) | 0.03-0.59 | 0.11±0.06 |  | 0.10±0.03 | 0.11±0.04 | 0.13±0.09 | p=0.15 | p=0.09 |
| ω-6/ω-3 | 2.50-28.28 | 11.72±3.90 |  | 13.09±4.25 | 11.15±3.01 | 10.10±3.49 | p<0.01 | p<0.01 |
| LA/ALA | 11.18-48.87 | 23.01±7.11 |  | 25.41±7.63 | 21.77±6.08 | 20.45±6.14 | p<0.01 | p<0.01 |
| AA/LA | 0.02-0.08 | 0.05±0.01 |  | 0.05±0.01 | 0.04±0.01 | 0.05±0.01 | p=0.13 | p=0.17 |
| DHA/ALA | 0.18-4.56 | 0.60±0.46 |  | 0.57±0.34 | 0.57±0.27 | 0.70±0.73 | p=0.43 | p=0.32 |
| DHA/AA | 0.23-4.55 | 0.60±0.43 |  | 0.52±0.23 | 0.61±0.24 | 0.73±0.73 | p=0.08 | p=0.05 |
| (EPA+DHA)/ALA | 0.22-5.00 | 0.71±0.51 |  | 0.67±0.38 | 0.67±0.31 | 0.81±0.80 | p=0.40 | p=0.29 |
| (EPA+DHA)/AA | 0.29-4.99 | 0.71±0.48 |  | 0.60±0.25 | 0.72±0.28 | 0.86±0.81 | p=0.05 | p=0.03 |
|  |  |  |  |  |  |  |  |  |

^a^Adjusted for maternal energy intake/maternal pre-pregnancy weight (kcal/day/kg); ^b^North includes breastfeeding mothers enrolled in Turin; ^c^Central includes breastfeeding mothers enrolled in Florence and Rome; ^d^South includes breastfeeding mothers enrolled in San Giovanni Rotondo and Palermo.

Abbreviations: ω-3, omega-3; ω-6, omega-6; AA, arachidonic acid; ALA, α-linolenic acid; DHA, decosahexaenoic acid; DPA, docosapentaenoic acid; EPA, eicosapentaenoic acid; LA, linoleic acid; MDS, Mediterranean diet score; MUFA, monounsaturated fatty acid; PUFA, polyunsaturated fatty acid; SD, standard deviation; SFA, saturated fatty acid.

**Supplementary table 3.** Mean and standard deviation of age, pre-pregnancy weight, and pre-pregnancy BMI of breastfeeding mothers according to the adherence to the Mediterranean diet score (expressed approximately in tertiles). MEDIDIET study, 2012-2014.

| **Variable** | **MDS** | | | **p-value**  **(ANOVA)** |
| --- | --- | --- | --- | --- |
|  | **0-3 points** | **4 points** | **5-8 points** |  |
|  | **mean±SD** | **mean±SD** | **mean±SD** |  |
|  |  |  |  |  |
| **Age (years)** | 32.75±3.78 | 31.77±4.54 | 34.19±3.65 | p<0.01 |
| **Pre-pregnancy weight (kg)** | 60.41±10.87 | 59.70±8.08 | 59.53±7.37 | p=0.76 |
| **Pre-pregnancy BMI (kg/m^2^)** | 22.34±3.51 | 22.18±3.33 | 22.28±2.78 | p=0.95 |
|  |  |  |  |  |
